# Evidence of neurodegeneration in autism spectrum disorder

**DOI:** 10.1186/2047-9158-2-17

**Published:** 2013-08-08

**Authors:** Janet K Kern, David A Geier, Lisa K Sykes, Mark R Geier

**Affiliations:** 1 Institute of Chronic Illnesses, Incorporation, Silver Spring, MD, USA; 2 University of Texas Southwestern Medical Center at Dallas, Dallas, TX, USA; 3 CoMeD, Incorporation, Silver Spring, MD, USA

**Keywords:** Autism spectrum disorder (ASD), Autism, Neurodegeneration, Regression, Neuronal cell loss, Microglia, Oxidative stress, Cytokines

## Abstract

Autism spectrum disorder (ASD) is a neurological disorder in which a significant number of children experience a developmental regression characterized by a loss of previously-acquired skills and abilities. Loss of neurological function in ASD, as observed in affected children who have regressed, can be explained as neurodegeneration. Although there is research evidence of neurodegeneration or progressive encephalopathy in ASD, the issue of neurodegeneration in ASD is still under debate. Evidence of neurodegeneration in the brain in ASD includes: (1) neuronal cell loss, (2) activated microglia and astrocytes, (3) proinflammatory cytokines, (4) oxidative stress, and (5) elevated 8-oxo-guanosine levels. The evidence from this review suggests that neurodegeneration underlies the loss of neurological function in children with ASD who have experienced regression and loss of previously acquired skills and abilities, and that research into treatments to address the issue of neurodegeneration in ASD are warranted.

## Introduction

The Diagnostic and Statistical Manual of Mental Disorders, 5th Revision (DSM-V) defines autism spectrum disorder (ASD) as a neurological disorder with a spectrum of qualitative impairments in social interaction, qualitative impairments in communication, and restricted and stereotyped patterns of behavior, interests, and activities [1]. ASD diagnoses disproportionately affect males (about 4–5 males per 1 female) and usually manifest in children before the age of 3 years [2,3]. Individuals diagnosed with an ASD are unable to learn from the natural environment in ways similar to neurotypical children. Most individuals diagnosed with an ASD show little interest in the world or people around them, and have a lifelong disability that affects the way a person comprehends, communicates, and relates to others. In addition, a recent study revealed individuals diagnosed with an ASD have a high prevalence of gastrointestinal disturbances, incontinence, sleep problems, eating disorders, hyperactivity, lethargy, sensory processing problems, anxiety/fear, behavior problems, and obsessive-compulsive behaviors [2]. Over the past two decades, the prevalence of individuals diagnosed with an ASD has risen dramatically [4]. Currently, at least 1 in 50 children are diagnosed with an ASD in the United States [5].

### Regression in ASD

In the context of neurodegeneration, the regression experienced by a significant number of children diagnosed with ASD who suffer a loss of previously-acquired skills is of particular interest [6-8]. Typically, regression in children diagnosed with an ASD is manifested by a loss of verbal, nonverbal, and social abilities [7-10]. The reported incidence of regression in individuals diagnosed with an ASD varies in different studies from 15% to 62% of cases [6,7,9,11,12].

Several studies have been undertaken to objectively evaluate the phenomenon of autistic regression early in life. For example, Werner and Dawson [13] evaluated home videotapes of children between their first and second birthday parties, including children diagnosed with an ASD, both with and without a reported history of regression, as well as typically developing children. Analyses revealed that infants diagnosed with an ASD with regression show similar use of joint attention and more frequent use of words and babble, compared with typical infants at 12 months of age. In contrast, infants diagnosed with an ASD with early onset of symptoms and no regression displayed fewer joint attention and communicative behaviors at 12 months of age. By 24 months of age, both groups of toddlers diagnosed with an ASD displayed fewer instances of word use, vocalizations, declarative pointing, social gaze, and orienting to name as compared with typically developing 24-month-olds.

Similarly, Ozonoff et al [14] evaluated the emergence of the early behavioral signs of an ASD diagnosis, including gaze to faces, social smiles, and directed vocalization coded from video and rated by examiners, by evaluating study subjects at 6, 12, 18, 24, and 36 months of age in a prospective longitudinal study. These investigators observed that the frequency of infant gaze directed to faces, shared smiles, and vocalizations to others were highly comparable between groups at 6 months of age, but these trajectories significantly declined over time in the group later diagnosed with an ASD. Group differences were significant by 12 months of age on most variables. These investigators concluded that their results suggest behavioral signs of ASD are not present at birth, as suggested historically by Kanner, but instead emerge over time through a process of diminishment affecting key social communication behaviors. Their results also suggest that more children with ASD may present with a regressive course than previously thought.

### Debate of neurodegeneration in ASD

Loss of neurological function in ASD, as observed in affected children who have regressed, can be explained as neurodegeneration or a type of progressive encephalopathy. Neurodegeneration is the progressive loss of structure or function of neurons. In diseases that are classically known as neurodegenerative, such as Parkinson’s disease (PD), Alzheimer’s disease (AD), or Huntington’s disease, early clinical symptoms manifest as neurological loss, which is also noted in children diagnosed with an ASD who have regressed. Courchesne et al [15] observed that there is evidence of progressive, age-related degeneration in the brains of individual diagnosed with an ASD. However, others have suggested that there is no evidence in autism of neurodegeneration [16]. Thus, to date, the issue remains under debate. The purpose of the present critical review is to provide an overview of the recent literature in neuroscience that provides important new insights into the phenomena of neurodegeneration in ASD.

### Neuronal cell loss and neurodegeneration

Many studies suggest ASD is characterized by neuronal cell loss [15]. At least seven post-mortem studies reported significant reductions in the number of cerebellar Purkinje cells (PCs) in the brains of individuals diagnosed with an ASD [17-23]. For example, Ritvo et al [23] found that the number of PCs in the vermis of the cerebellum were approximately 15 standard deviations below the mean and approximately 8 standard deviations below the mean bilaterally in the cerebellar hemispheres, in the individuals diagnosed with an ASD in comparison to neurotypical controls. According to a postmortem study by Whitney et al [24] PCs were generated, migrated to their proper location in the PC layer but subsequently died.

Other studies demonstrate that the neuronal pathology found in the brain of individuals diagnosed with an ASD is suggestive of a neurodegenerative process. After examining the brains of individuals diagnosed with an ASD, Vargas et al [22] reported degeneration in PCs and also microglial activation (microglial activation will be discussed further in the next section). Bailey et al [20] reported the PC loss was associated with gliosis, indicating that these changes are acquired rather than neurodevelopmental in nature [25] [Gliosis is proliferation of neuroglial tissue that follows neural damage] [26].

Other studies have also observed that fewer neurons are found in other areas of the brain in individuals diagnosed with an ASD when compared to neurotypical subjects. Neuronal numbers were found to be fewer in the amygdala of individuals diagnosed with an ASD than controls [27]. Other investigators observed that neurons in the fusiform gyrus were also fewer (and smaller) in individuals diagnosed with an ASD as compared to controls. Specifically, these significant reductions in neuron densities were found in layer III, total neuron numbers in layers III, V and VI, and mean perikaryal volumes of neurons in layers V and VI in the fusiform gyrus [28]. Postmortem studies also show fewer pyramidal cells in the brains of individuals diagnosed with an ASD when compared to controls [29].

### Microglia activation and neurodegeneration

Microglial reactivity is commonly noted in neurodegenerative diseases [30]. Microglia, a type of glial cell, are the resident tissue macrophage of the central nervous system (CNS). Microglia promote inflammation in infected or damaged tissue and are important for maintaining homeostasis in non-infected regions [30]. Microglia act as key cellular mediators of the neuroinflammatory processes and are associated with the pathogenesis of many neurodegenerative and brain inflammatory diseases and disorders, such as AD, PD, stroke, spinal cord injury, encephalitis, and multiple sclerosis (MS) [30,31].

Although the role of microglia in neurodegeneration is not entirely understood, evidence indicates that microglia can become transiently activated to an amoeboid phenotype responsible for the phagocytosis of living neurons [32,33]. In addition, particularly in long-term neuroimmune activation, microglia produce cytokines that are toxic to neurons, and this neurotoxicity plays a potential role in neurodegeneration [34]. With sustained microglial activation, collateral neural damage of healthy brain tissue can result [35]. It is interesting to note that Nam et al [36] found (using a model of mitochondrial membrane potential loss) there was only 25% cell loss in SH-SY5Y (SH) neuronal mono-cultures, but that 85% neuronal loss occurred when neurons were co-cultured with BV2 microglia. These investigators reported that SH neurons overexpressing uncoupling protein 2 exhibited an increase in neuron-microglia interactions, which represented an early step in microglial phagocytosis of neurons.

Sometimes neurodegeneration involves degeneration of presynaptic terminals prior to the loss of the cell body. There is some debate as to the involvement of microglia in synaptic stripping and synapse degeneration [37].

Numerous recent studies provide evidence that individuals diagnosed with an ASD suffer from an ongoing neuroinflammatory process in different regions of the brain involving microglial activation [22,38-43]. Evidence from post-mortem brain tissue documents activated microglia and astrocytes [22,38,39].

Vargas et al [22] observed that among the brain regions studied, the cerebellum showed the most neuroglial responses in individuals diagnosed with an ASD. They stated that the selective process of neuronal degeneration and neuroglial activation appears to occur predominantly in the PC layer and the granular cell layer of cerebellum and that these findings are consistent with an active and ongoing postnatal process of neurodegeneration and neuroinflammation. They also stated that the proinflammatory chemokine, monocyte chemotactic protein-1 (MCP-1), was consistently elevated in the brain regions studied in individuals diagnosed with an ASD, and that increased expression of MCP-1 may have relevance to the pathogenesis of ASD because its elevation in the brain is linked to microglial activation, and perhaps, to the recruitment of monocytes/macrophages to areas of neurodegeneration in the cerebellum. Importantly, these investigators concluded that their observations are similar to findings in other neurodegenerative diseases in which elevation of MCP-1 is associated, such as human immunodeficiency virus (HIV) dementia, amyotrophic lateral sclerosis (ALS), and multiple sclerosis (MS).

Microglial activation is found in the cerebrum as well as the cerebellum of individuals diagnosed with an ASD. Tetreault et al [43] immunocytochemically identified microglia and stereologically quantified microglial densities in the fronto-insular and visual cortex from autopsies of the brain in individuals diagnosed with an ASD in comparison to matched controls. They observed increased densities of microglia in both cortical areas in the brains of individuals diagnosed with an ASD as compared to controls. Suzuki et al [42] used positron emission tomography (PET) and a radiotracer for microglia to identify brain regions associated with excessively activated microglia in the whole brain, in individuals diagnosed with an ASD as compared to controls. They determined that increased microglial activation was present in the: cerebellum, midbrain, pons, fusiform gyri, and the anterior cingulate and orbitofrontal cortices, in individuals diagnosed with an ASD in comparison to controls. The investigators also observed, in accord with the observations made by Vargas et al [22], that the area with the most prominent increase was the cerebellum in individuals diagnosed with an ASD in comparison to controls.

### Proinflammatory cytokines and neurodegeneration

Activated microglia can release a number of potentially neurotoxic substances, such as reactive oxygen species, nitric oxide, and various proinflammatory cytokines, and evidence implicates neuroinflammation and overproduction of proinflammatory cytokines as a contributor to pathophysiology of chronic neurodegenerative disorders [44]. Proinflammatory cytokines are found in chronic neurodegenerative disorders such as AD, PD, and MS.

Studies show elevated proinflammatory cytokines in the brains and spinal cords of individuals diagnosed with an ASD. Li et al [45], for example, showed that proinflammatory cytokines (tumor necrosis factor (TNF)-α, interleukin (IL)-6 and granulocyte-macrophage colony-stimulating factor (GM-CSF), Th1 cytokine (interferon (IFN)-γ) and chemokine (IL-8) were significantly increased in the brains of individuals diagnosed with an ASD compared with controls. A study by Vargas et al [22] demonstrated tumor growth factor-β1, derived from neuroglia, was significantly increased in the middle frontal gurus (MFG) of individuals diagnosed with an ASD, while MCP-1, IL-6 and IL-10 were increased in the anterior cingulated gurus (ACG), in comparison to controls. In addition, using a protein array approach, Vargas et al [22] also found that MCP-1, IL-6, IL-8 and IFN-γ were significantly increased in the cerebrospinal fluid (CSF) in individuals diagnosed with an ASD in comparison to controls. Chez et al [46] also observed that there was a significant increase in TNF-α in the CSF of individuals diagnosed was an ASD in comparison to the concurrent serum levels and that the magnitude of the difference was significantly higher than that observed for other diseases.

### Oxidative stress and neurodegeneration

Over production of reactive oxygen species (ROS) or oxidative stress is a central feature of neurodegenerative disorders [47]. Postmortem brain tissues from individuals diagnosed with neurodegenerative disorders, including PD, AD and ALS, display increased levels of ROS in affected brain regions [48].

Free radicals and other ROS are produced in all species. Any free radical involving oxygen can be referred to as ROS, e.g. nitric oxide (NO). Free radicals and other ROS are unstable atoms, molecules, or ions with unpaired electrons. They are harmful because the unpaired electron oxidatively reacts with other ions and molecules, or, by “stealing” an electron from other molecules, to pair that electron. This produces disruption to other molecules and damage to cells [49]. One of the main problems is that ROS “steal” electrons from lipid membranes (the cell membrane of most living organisms is made of a lipid bilayer). The oxidative degradation of the lipid membrane is referred to as lipid peroxidation. Lipid peroxidation results in loss of membrane integrity and fluidity, which ultimately leads to cell death [50,51]. ROS also react with proteins and nucleic acids which can lead to cell death via apoptosis or necrosis [52].

Under normal conditions, a dynamic equilibrium exists between the production of ROS and the antioxidant capacity of the cell [53]. Oxidative stress occurs when there is an imbalance between free radicals and the ability to neutralize them (i.e. an excess of pro-oxidants, a decrease in antioxidant levels, or both). The brain is highly vulnerable to oxidative stress due to its limited antioxidant capacity, higher energy requirement, and higher amounts of lipids and iron [54].

In ASD, several post-mortem studies reveal that affected areas of the brain in individuals diagnosed with an ASD showed accelerated cell death under conditions of oxidative stress [55-61]. For example, Lopez-Hurtado and Prieto [55] found that the density of lipofuscin, a matrix of oxidized lipid and cross-linked protein that forms as a result of oxidative injury in the tissues, was observed to be greater in cortical brain areas concerned with communication in individuals diagnosed with an ASD than in controls. As mentioned earlier, individuals diagnosed with an ASD typically lose speech and language abilities at the time of regression.

Other studies have found elevated oxidative stress markers in areas of the brain associated with ASD symptoms. Sajdel-Sulkowska et al [59]. reported that the brain regions with the highest levels of the oxidative stress marker, 3-nitrotyrosine (3-NT), were in the orbitofrontal cortex, Wernicke's area, cerebellar vermis, cerebellar hemisphere, and pons (brain areas associated with the speech processing, sensory and motor coordination, emotional and social behavior, and memory) in individuals diagnosed with an ASD.

In two other studies by Sajdel-Sulkowska et al [57,58], they found elevated 3-NT and neurotrophin-3 (NT-3), markers of oxidative stress, in the cerebellum of individuals diagnosed with an ASD in comparison with controls. Evans et al [56] found elevated oxidative stress markers in the brains of individuals diagnosed with an ASD by evaluating the oxidative stress metabolites of carboxyethyl pyrrole (CEP) and iso[4]levuglandin (iso[4]LG)E2-protein adducts in cortical brain tissues. These investigators reported that the striking thread-like pattern appears to be a hallmark in the brains of those diagnosed with an ASD, as it was not seen in any neurotypical brains, young or aged, used as controls for the oxidative assays. Chauhan et al [60] compared DNA oxidation and glutathione redox status in postmortem brain samples from the cerebellum and frontal, temporal, parietal and occipital cortex from individuals diagnosed with an ASD and age-matched neurotypical controls. These investigators reported that DNA oxidation was significantly increased by two-fold in the frontal cortex, temporal cortex, and cerebellum in individuals diagnosed with an ASD compared to controls. Moreover, the levels of reduced glutathione were significantly reduced, and the levels of oxidized glutathione were significantly increased, in samples of the cerebellum and temporal cortex from individuals diagnosed with an ASD as compared to the corresponding levels in the control brain samples. Earlier, Chauhan et al [61] found a significant increase in the levels of lipid hydroperoxides, an oxidative stress marker, in the cerebellum and temporal cortex in individuals diagnosed with an ASD as compared to controls.

### 8-oxo-guanosine (8oHdG) and neurodegeneration

8oHdG is an RNA oxidative damage marker that can be used to assess oxidative stress that is found in the brain in neurodegenerative disease [62]. Urinary 8OHdG has been used successfully to measure brain damage and degeneration, showing a significant correlation (r = 0.87, p < 0.01) with serum S100beta values, which are already used to measure brain damage [63]. Sajdel-Sulkowska et al [58] conducted a study where they examined oxidative damage in the cerebellum of those individuals diagnosed with an ASD by measuring 8OHdG. The authors found that cerebellar 8OHdG showed an upward trend toward higher levels with an increase of 63.4% observed in those individuals diagnosed with an ASD in comparison to controls.

## Conclusion

To date, the etiology of ASD remains under debate. There are, however, many studies that suggest toxicity in children with ASD. A recent study conducted by the Harvard School of Public Health [64], for example, found that perinatal exposures to the highest versus lowest quintile of diesel, lead, manganese, mercury, methylene chloride, and an overall measure of metals were significantly associated with ASD, with odds ratios ranging from 1.5 (for overall metals measure) to 2.0 (for diesel and mercury). Similarly, Windham et al [65] examined possible associations between ASD and environmental exposures in 284 children with ASD and 657 controls, born in 1994 in the San Francisco Bay area. They found that the individual compounds that contributed most to these associations included mercury, cadmium, nickel, trichloroethylene, and vinyl chloride, with the single largest risk factor being mercury. These findings that show an association between toxic exposure and ASD, particularly mercury exposure, are corroborated by many other studies [66-69]. In addition, studies show biomarkers of susceptibility to toxins, such as reduced plasma and brain tissue glutathione levels in ASD. Importantly, polymorphisms in glutathione-related genes modify mercury concentrations and antioxidant status in human subjects environmentally exposed to mercury [70]. Yet, as mentioned earlier, the World Health Organization states that ASD cannot be associated with toxic exposure because there is no evidence of neurodegeneration in ASD.

However, the research examined in this critical review provide compelling evidence for neurodegeneration as the underlying mechanism for the loss of neurological function in individuals diagnosed with an ASD who have regressed and lost previously acquired skills and abilities. As with other neurodegenerative diseases, the evidence of neurodegeneration in the brain in ASD includes: (1) neuronal cell loss, (2) activated microglia and astrocytes, (3) proinflammatory cytokines, (4) oxidative stress, and (5) elevated 8-oxo-guanosine levels. In addition, areas of the brain that have shown evidence of neurodegeneration correlate to areas known to be affected in ASD, including areas of speech and socialization.

The current treatments of choice used in individuals diagnosed with an ASD include educational interventions such as applied behavioral analysis (ABA) and/or psychoactive drugs such as Risperdal. Neither of these types of treatments addresses the apparently common issue of neurodegeneration present in the brain of individuals diagnosed with an ASD. Future research should be undertaken into treatments that address issues of neurodegeneration in ASD. The evidence also suggests that regressive autism should be included in the same category as other neurodegenerative diseases.

## Competing interests

The authors declare that they have no competing interests.

## Authors’ contributions

JK was the main writer, DG was wrote and edited, LS reviewed, analyzed manuscript structure and edited, MG evaluated and reviewed the ideas and science. All authors read and approved the final manuscript.
